# Identifying strategies to promote team science in dissemination and implementation research

**DOI:** 10.1017/cts.2019.413

**Published:** 2019-09-09

**Authors:** Gregory A. Aarons, Kendal Reeder, Christopher J. Miller, Nicole A. Stadnick

**Affiliations:** 1 Department of Psychiatry, University of California San Diego, La Jolla, San Diego, CA 92093-0812, USA; 2 Child and Adolescent Services Research Center, San Diego, CA 92123, USA; 3 Center for Healthcare Organization and Implementation Research (CHOIR), VA Boston Healthcare System, Boston, MA 02130, USA; 4 Department of Psychiatry, Harvard Medical School, Boston, MA 02115, USA

**Keywords:** Team science, dissemination, implementation, nominal group technique, cross-disciplinary

## Abstract

**Introduction:**

Scientific endeavors are increasingly carried out by teams of scientists. While there is growing literature on factors associated with effective science teams, little is known about processes that facilitate the success of dissemination and implementation (D&I) teams studying the uptake of healthcare innovations. This study aimed to identify strategies used by D&I scientists to promote team science.

**Methods:**

Using a nominal group technique, a sample of 27 D&I scholars responded to the question, “What strategies have you or others used to promote team science?” Participants were asked to individually respond and then discuss within a small group to determine the group’s top three strategies. Through a facilitated consensus discussion with the full sample, a rank-ordered list of three strategies was determined.

**Results:**

A total of 126 individual responses (*M* = 9; SD = 4.88) were submitted. Through small group discussion, six groups ranked their top three strategies to promote team science. The final ranked list of strategies determined by the full sample included: (1) developing and maintaining clear expectations, (2) promoting and modeling effective communication, and (3) establishing shared goals and a mission of the work to be accomplished.

**Conclusions:**

Because of its goal of translating knowledge to practice, D&I research necessitates the use of team science. The top strategies are in line with those found to be effective for teams in other fields and hold promise for improving D&I team cohesion and innovation, which may ultimately accelerate the translation of health innovations and the improvement of care quality and outcomes.

## Introduction

Across the globe, a focus on teamwork and collaboration in the workplace is on the rise.^1^^,^^2^ This growing trend is present in a variety of settings, including scientific research contexts.^3^ Team research efforts, referred to as team science, involve multiple scientists who carry out interdependent tasks to achieve a goal.^4^ Compared to single investigators, science teams not only produce more academic publications, but also achieve higher impact factors, are cited more frequently, and are more likely to discover breakthroughs.^3^^,^^5^ While scientific collaborations are often unidisciplinary in nature (i.e., within the same field), scientists are increasingly working in cross-disciplinary teams that bridge two or more fields and/or patients, community stakeholders, and other relevant stakeholders. However, engaging researchers and collaborators in implementation science teams and fostering the development of these teams can be a slow process.^4^

Cross-disciplinary collaborations have been characterized as multidisciplinary, interdisciplinary, or transdisciplinary.^6^ In a multidisciplinary team, researchers from different disciplines work within their respective fields and then apply their findings to a common research goal. For example, an epidemiologist might conduct a study while an anthropologist might address the same issues from a qualitative inquiry perspective, with their findings integrated post hoc. Interdisciplinary research involves more integration, such that researchers from separate fields (e.g., anthropology, medicine, social work) interactively work together to address a common problem (e.g., access to high-quality healthcare). Transdisciplinary research, the most collaborative form, involves a synthesis of distinct fields to develop models that transcend a single discipline and result in a hybrid discipline. For example, neuroscience, bioengineering, and human–computer interaction are hybrid disciplines that evolved from transdisciplinary efforts.^7^ We use the term cross-disciplinary to refer to science teams that collaborate at any of these levels.

Because cross-disciplinary teams may foster greater potential for innovation and creativity,^8^^,^^9^ they are especially important for tackling complex public health issues.^10^^–^^12^ As such, recent decades have seen a substantial increase in the amount of funding provided by both private foundations and federal agencies for team-based research efforts.^13^ For example, National Institutes of Health (NIH) awards to projects with multiple principal investigators, first introduced in 2006, accounted for nearly 20% of all major grants funded in 2013.^13^^,^^14^ To further facilitate team science, NIH has established multiple cross-disciplinary programs and research centers.^15^^,^^16^ Team-based research efforts increasingly involve scientists from different organizations^13^; multi-university collaborations have progressively become more common, and there have been recent calls to extend the scope of science team membership beyond academia.^17^^–^^19^ Because many cross-disciplinary research initiatives ultimately aim to apply research findings to real-world settings to combat widespread public health issues,^12^^,^^20^^,^^21^ collaborating with public stakeholders in team-based research efforts is a promising strategy to facilitate translation of these initiatives.^22^^,^^23^

Moving from research to practice to improve health services and population health is an objective of the new science of dissemination and implementation (D&I).^24^ There is a well-documented lag between the development of scientific evidence (e.g., new medications, psychosocial interventions) and their broad use in practice. This has resulted in a lag of roughly 17 years in the translation pipeline and then only about 14% of original research goes to the benefit of patient care.^25^ Teams are critical for D&I science, which naturally facilitates engagement between scientists and stakeholders (e.g., researchers within other disciplines, policymakers, community members) to accelerate the translation of new and effective evidence-based practices.^26^^,^^27^ Cross-disciplinary D&I science teams have the potential to expedite the translation process through having the most relevant and innovative perspectives come together to determine the most efficient and effective ways to move research to practice. While there are challenges associated with co-production in science teams (e.g., disagreements, professional costs),^28^ research has described the richness and complexity of collaborative processes while also identifying multiple benefits that outweigh the challenges.^29^ The requisite for teams to produce translatable outcomes, coupled with increased calls for the use of team science in D&I settings,^30^^–^^33^ creates a need for evidence-based strategies for facilitating successful D&I collaborations and synergistic transdisciplinary teams.^34^

Rich findings from research on team functioning suggest strategies for creating effective cross-disciplinary science teams.^35^ This literature highlights strategies that enable successful teams, such as communication, the development of shared mental models (i.e., knowledge structures), trust, and shared leadership.^36^^–^^40^ While team effectiveness findings have typically been applied across settings, contextual factors may limit their generalizability.^11^ Many findings come from studies of randomly composed teams in laboratory settings, where tasks and performance measures tend to be narrow. As such, literature on successful teams in real-world settings is on the rise, allowing strategies to be tailored to teams based on their setting and goals.^35^ For example, studies on cross-disciplinary healthcare teams have found that team processes associated with team success include quick learning, intent listening, speaking up, and psychological safety.^41^^,^^42^

The increasing calls for team science, however, have outpaced research on how to promote effective science teams. There are a number of questions relevant to science teams that have not been addressed, such as the effects of research structures and funding mechanisms on team functioning.^13^ These questions and others are beginning to be addressed by the science of team science field, which aims to identify and understand strategies that are associated with successful science teams. Science of team science research has begun to uncover team processes that lead to effective team science, such as learning from other members and communicating face-to-face.^34^ Yet amidst calls for the greater use and study of team science in D&I contexts,^30^^–^^33^^,^^43^ no studies have investigated strategies that facilitate success in D&I science teams.^44^ The aims of D&I science teams, which may include implementing evidence-based practices with fidelity,^45^ sustaining these practices in community settings,^22^^,^^46^ or engaging public stakeholders in the research process,^47^ introduce unique challenges not faced by cross-disciplinary teams that operate solely within academia.^18^^,^^44^ The objective of this study was to identify and rank strategies to promote team science among scholars engaged in D&I research.

## Methods

### Procedures

The nominal group technique (NGT) was used in this study. The NGT is a commonly used group consensus method in health services research that involves a facilitated, multistep structured group process to generate and prioritize responses to a stimulus question among a panel who have expertise in a focal area,^48^^–^^51^ without relying on an *a priori* established conceptual model that might inadvertently bias results. This approach was used for pragmatic reasons (e.g., minimal preparation, only one meeting needed) and for fit with the goal of the meeting (i.e., to identify strategies used by D&I researchers to promote team science). In addition, we wanted to capitalize on the unique opportunity to learn from a diverse group of multidisciplinary researchers engaged in D&I research. We applied the recommendations to ensure methodological rigor when using consensus group methods to report the steps and results from the NGT approach used in this study.^52^

We used a modified NGT process that proceeded in the following stages. First, the facilitator (GAA) presented the nominal question: What strategies have you or others used to promote team science? Participants were first asked to generate ideas and write down their responses to this question individually. They were also asked to rank order their top three ideas. Next, small groups (consisting of 3–6 individuals) were formed and participants were asked to take turns sharing all of their individual responses with the group, to compile strategies within the group, and to rank order the top three strategies agreed upon by their small group (round 1). Following the first two rounds, the facilitator requested that each small group, in turn, report out their ratings and these were written out on a flip chart for all to view and consider (round 2). The facilitator then led all participants in a full group consensus ranking process to determine the top three strategies (round 3). After each round, the facilitator summarized responses and elicited participant confirmation of the summaries. Collective group consensus was defined as agreement indicated by verbal and/or nonverbal approval.

### Setting

This study was conducted during the weeklong in-person training component of the National Institute of Mental Health (NIMH)/Veterans Affairs Health System (VA)/National Institute on Drug Abuse (NIDA)-sponsored Implementation Research Institute (IRI) on June 11, 2018 at the Knight Executive Education and Conference Center and was hosted by the George Warren School of Social Work and the Institute for Public Health at Washington University in St. Louis, Missouri. Data were collected as part of an hour-long interactive activity focused on team science and working on cross-disciplinary teams within D&I research. This study was approved by the Institutional Review Board of the University of California, San Diego as exempt research.

### Participants

Twenty-one IRI fellows and six core faculty (*n* = 27) attended the presentation. The IRI is a 2-year interdisciplinary training program that provides in-depth training in D&I science through an annual learning collaborative, individualized mentoring, and a mentoring network of faculty and fellows to advance the research and careers of implementation science investigators. Participants were predominantly female (67%, *n* = 18). In terms of professional characteristics, the majority of participants had doctoral (i.e., PhD or equivalent) research degrees (82%, *n* = 22) and the remaining participants had medical degrees (19%; *n* = 5). There was a range of disciplines represented across the participants: Psychology (37%, *n* = 10), Social Work (7%, *n* = 2), Medicine (7%, *n* = 2), Psychiatry (7%, *n* = 2), Pediatrics (7%, *n* = 2), Public Health (7%, *n* = 2), Engineering (7%, *n* = 2), Anthropology (4%, *n* = 1), Sociology (4%, *n* = 1), Organizational Behavior and Management (4%, *n* = 1), Education (4%, *n* = 1), and Health and Public Policy (4%, *n* = 1). Participants were conducting implementation research in both domestic and international (i.e., low- and middle-income countries) settings and in a variety of healthcare contexts (e.g., primary care, mHealth, public systems, organizational level, schools, community mental health, prisons, Federally Qualified Health Centers). See Table 1 for participant characteristics.

**Table 1.** Sample characteristics

Demographic or professional characteristic	% (*n*)
Gender (female)	67 (18)
Education	
PhD or equivalent	82 (22)
MD	19 (5)
Primary discipline	
Psychology	37 (10)
Medicine	7 (2)
Psychiatry	7 (2)
Public Health	7 (2)
Engineering	7 (2)
Anthropology	4 (1)
Sociology	4 (1)
Organizational behavior and management	4 (1)
Education	4 (1)
Health and Public Policy	4 (1)

Note: *n* = 27

The facilitator (GAA) requested to collect individual and group written responses from those attendees who were willing to share their data. The NGT process typically includes 5–12 members, so our sample size of 27 exceeds this standard.^52^ Anonymity of participants was maintained by ensuring that no personally identifying information could be linked to a response, and no additional qualitative data were collected.

### Data Analysis

Descriptive statistics were used to summarize the individual data and aggregated rankings. Data analysis and reduction was conducted as part of the NGT process so that data aggregation and rankings were complete at the end of the participatory group exercise.

## Results

During the NGT exercise, in round 1, individual participants were asked to report, from their own written list, their top three strategies. In round 2, each of six small groups considered all of the individual written responses of all group members and reported out to the full group the top three strategies agreed upon within their small group. In round 3, group consensus was achieved from all small groups. After the third round, 14 of the 27 participants (response rate = 52%) submitted their individual written responses from round 1 to the research team for a total of 126 responses. The average number of individual responses was 9.00 (SD = 4.88; range = 4–18).

The three strategies from each of the six groups identified through the consensus process, along with their definitions, are reported in Table 2. Through full group discussion, the top strategies endorsed by each small group were consolidated into four primary categories. The categories were: (A) a focus on clear expectations, responsibilities, and well-defined roles of team members, (B) promoting effective communication, which includes respectful and assertive communication styles between team members and leadership, (C) fostering a culture of mutual respect and trust, and (D) developing a shared mission and/or set of goals. All six small groups submitted at least one strategy related to Category A, four of the small groups submitted at least one strategy related to Category B, four of the small groups submitted at least one strategy related to Category C, and three of the small groups submitted a strategy related to Category D. As shown in Table 3, the full group’s consensus process (i.e., all participants) identified the top three strategies for promoting team science in implementation and their respective ranks. Through the facilitated group consensus discussion (round 3), the final rank-ordered list of strategies for promoting team science were: (1) developing and maintaining clear expectations, (2) promoting and modeling effective communication, and (3) establishing shared goals and a mission of the work to be accomplished. Individual participant responses are provided in Table 4.

**Table 2.** Top three strategies identified by small groups and rank ordered within group

Group	Rank	Strategy	Category
1	1	Relationships/trust	C
	2	Clear expectations	A
	3	Communication frequency, shared language	B
2	1	Shared goals/mission	D
	2	Role clarity	A
	3	Psychological safety	C
3	1	Consensus building	D
	2	Clear responsibilities	A
	3	Open and efficient	B
4	1	Identity roles and expertise	A
	2	Positive reinforcement	B
	3	Modeling respect	C
5	1	Clear expectations/roles	A
	2	Shared mental models and goals	D
	3	Nontraditional perspective	C
6	1	Leadership modeling assertive communication	B
	2	Dedicated support to organize	A
	3	Seminar to demonstrate skills and capacity to each other	B

**Table 3.** Top three strategies identified in full group consensus ranking process

Strategy	Rank	Definition
Clear expectations/roles	1	Defining roles and setting expectations
Effective communication	2	Effectively and efficiently communicating
Shared goals/mission	3	Developing shared mission and goals

**Table 4.** Strategies identified by individuals

Individual 1	Individual 2	Individual 3	Individual 4	Individual 5
Clear roles and contributions, expectations at outset	Actively seeking expertise outside of my own	Recruit clinical co-investigators and multiple PI’s for grant applications	Shared vision or mission, stress importance and passion	Engage investigators in other disciplines
Set frequency and schedule ad hoc as necessary	Ensure a shared mental model of the team goals so everyone understands the end points, developing team strategy	Reach out beyond institution to engage methods experts/statisticians	Clarity in role expectations	Clear communication of deadlines and request what you need of them
Impose deadlines, even if need to be changed	In-person meetings both formal & informal	Engage qualitative researchers	Clarity in measuring progress	Explain reasons for your decisions and speak your point
Establish meeting agendas	Web-based meeting sometimes	Hire nontraditional people (geologist, biochemist)	Atmosphere of trust and safety, fun	
Establish specific assignments on subtasks	Balanced relationships: I help you with x, you help me with y	Read broadly to search for the best ideas to apply to your research problem		
Set clear goals and subgoals				
Evaluate team processes and goals periodically				

Individual 6	Individual 7	Individual 8	Individual 9	Individual 10
Clear delineation of roles and responsibilities	Fun team building activities	Regular meetings (in person or virtual)	Clear delegation of responsibilities/role clarity	Find mutual goal (consensus building)
Getting everyone’s input into goals, tasks (buy-in)	Integrated vertical teams	Transparency in approach	Open/effective communication	Find outcome for motivation for involvement
Making sure everyone has something to lead	Mentorship from those who just completed task	Accountability to each other	Frequent feedback to the team	Tell people who work for you what to do
Team building/socializing (lunch, etc.)	Cloud-based/interactive editing/scheduling	Reaching out to other disciplines to learn new approaches	Supportive of one another	Convince others to join team (inform)
Trying to head off potential conflicts: discuss who is PI, who is lead on paper early	Periodic evaluation	Listen	Clear leadership structure	Conciliation of adaptation
Tailor work assignments to team members’ strengths and preferences	Regular meetings – cancel when none needed	Reflection on processes	Monitor for burnout/practice helping behaviors	Guilt/persistence
Always give credit for accomplishments to the whole team, but still acknowledge those who took a leadership role	Communication about authorship in advance	Feedback/assessment		
If there’s conflict, manage it right away – don’t hope it will go away	Feedback mechanisms for leadership	Goals		
Give people autonomy to do their tasks – don’t micromanage				
Open door policy in terms of questions help				

Individual 11	Individual 12	Individual 13	Individual 14
Communication lines	Develop common vocabulary	Inclusion in future work	Multidisciplinary
Identifying roles/areas of expertise	Increasing meeting frequency	Interactive online meetings	Team meetings with reflection
Developing areas of expertise	Organization structure/hierarchy	Read management books	Periodic research meetings (across teams)
Sectioning/tailoring requests for feedback	Shared students – shared goals	Background readings in one another’s fields	Agendas, timelines, to-do lists
Positive reinforcement/building up	Develop common goals	Modeling of respect by leader (multidisciplinary)	Spin off smaller groups
Setting ground rules	Increasing understanding of each other’s roles	In-person meetings	Clarify hierarchies, roles, responsibilities
Social engagement	Equality in meeting location	Shared language	Establish paper proposal templates
Creating team layers	Listening – active	Shared goals and being reminded of them	Writers’ week
Supporting/shifting leadership roles	Assessment and feedback	Conference calls	Video/telephone meetings and face-to-face when possible
Sharing resources	Information sharing	Agendas sent in advance	Bring in outside expertise as needed
Identifying common goals	Clear delegation of responsibilities	Emails	Relational coordination: trust, respect, psychological safety, communication
Modeling of respect	Communication and feedback	Letting different people lead each call	Shared goals/mission
Interactive online meetings	Support	Flexibility with scheduling	
Having agendas set in advance	Role delineation	Senior to junior support with working with different fields	
Documentation of team responsibilities + revisiting	Getting input on one’s goals and tasks	“Translator” role	
Show by example	Develop common goals		
Less hierarchical	Increasing understanding		
Make sure align incentives			

## Discussion

We examined factors recommended to facilitate team science among a cohort of D&I researchers, including experienced faculty and early- to mid-career fellows of the NIMH/VA/NIDA-sponsored IRI. The top three strategies for fostering team science in D&I (by rank order) endorsed by the group were (1) setting clear expectations and roles in conducting research, (2) promoting and modeling effective communication, and (3) establishing shared goals and mission. These findings are broadly consistent with promoting team science in other fields,^34^^,^^35^^,^^37^ and with findings and conceptual models regarding team building more broadly.^53^^,^^54^ There are several possible reasons that these aspects of team functioning were seen as particularly relevant by our respondents. First, D&I science typically brings together disparate fields such as clinical services, implementation, organization, policy, and economics – in fact, many implementation science questions are impossible to answer *without* involving these diverse viewpoints. Each of these fields may bring different expectations regarding what research goals are most important and how best to communicate within the team. Thus, there is a need to reformulate research training to foster a more interprofessional perspective in favor of focusing too much on one’s own discipline.^55^ Such an approach could lead to more professional empathy regarding other perspectives in team science. Second, because D&I science is a relatively new field, there will likely be variability among team members regarding the breadth and depth of familiarity with D&I concepts, frameworks, research designs, methods, and measures. This also suggests that clear communication may be especially important for D&I science teams. While the NGT resulted in the top three recommended strategies, it is important to acknowledge that there were many other recommendations from one or more of the six small groups that should also be considered. For example, relationships, psychological safety, and trust are important in research and also important in collaborative and mentoring relationships. Developing consensus while modeling respect and developing a sense of psychological safety can foster cross-disciplinary team science where there is a need for respect of differing perspectives and values. We also recommend that D&I research aspires to the level of “transdisciplinary” research to support effective collaboration and to promote synergy and innovation.

This recommendation naturally begs the question: What strategies might be used to develop transdisciplinary teams that will be innovative, effective, and productive in the field of D&I science? The answers are beyond the purview of the present study; however, we suggest that effective leadership and development of collaboration and mentoring networks can help to achieve these goals. These were identified in the NGT process and warrant further consideration. For example, the Leadership and Organizational Change for Implementation intervention,^56^ while designed to create a climate for implementation of evidence-based practices, could be adapted to create a climate for team science in academic settings and in community academic partnerships.^57^

There are many frameworks and theories that help to organize and describe implementation determinants and processes.^58^^,^^59^ The Exploration, Preparation, Implementation, Sustainment (EPIS) framework describes outer (e.g., health system) and inner (i.e., organization) factors that might impact implementation as well as the four phases of the implementation process.^60^^,^^61^ It is useful to consider how multidisciplinary teams of different configurations might be useful at different phases of the implementation process and also for understanding outer and inner context factors. For example, including an organizational psychology perspective can be useful in understanding operations in hospitals, clinics, or teams. Much of this work can also be considered to occur in the Exploration and Preparation phases of the EPIS framework. For example, the EPIS processes of collaboration or co-production as “bridging factors” that span outer and inner contexts can be considered to be active in bringing together diverse stakeholders including researchers and community or health system partners.^61^ Such partnerships and collaborations can help to make implementation science more immediately relevant both theoretically and practically.

The call for team science is becoming the norm for research funders and academic institutions. For D&I research, team science and the need for collaboration to share and integrate perspectives, ideas, and ideals are essential and may be even more critical than in other fields. This is a function of the broad purview of concepts and approaches and crossing-cutting issues and contexts in D&I. These include different disease and health promotion areas, patient and service populations, service systems and settings that span geographical contexts (e.g., different continents, countries, municipalities), and technology platforms (e.g., eHealth, mHealth) where implementation occurs. Because of the inherently interdisciplinary and collaborative nature of the field of D&I science, it is imperative that we understand how to promote appropriate team science. This includes building teams with needed expertise and skills for professional empathy to both appreciate and apply the perspectives, theories, and methods of others working together in pursuit of solving D&I issues and advancing the field.

Even the process of funding invokes the need for team science. For example, in the USA, there are formal calls for D&I research that involves over 17 of the institutes within the NIH. In the Dissemination and Implementation Research in Health (DIRH) review committee, tasked with the scientific evaluation of D&I research, proposal members may have specific expertise in a given disease or public health category (e.g., cancer, addiction, mental health). However, the common language, concepts, and approaches of proposals being evaluated is that of D&I. The DIRH review panel itself could be considered an interdisciplinary evaluation team. While this is a promising approach to scientific review of these types of applications, there are still different and varied perspectives depending on disease and contextual issues (e.g., implementation in low- and middle-income countries) that require more effective team science in the conduct of science and, as in this committee, the evaluation of the science.

The findings presented in this study raise questions about the ease and difficulties of teams in implementation science. For example, the top-ranked strategy was related to clear expectations, responsibilities, and well-defined roles of team members. Implementing this strategy may present challenges where there are strong opinions or preference for leadership roles versus team member roles. Promoting effective communication can be dependent on the ability of leaders to engender respectful communication among team members, and the development of mutual respect and trust to create a shared mission and/or set of goals. In addition to the current study results, more detail about potential barriers to team science can be garnered from “lessons learned” from a team science project addressing multiple chronic health conditions.^62^ Reflecting on that process, the authors identified the need for funding for effective infrastructures to foster team science, taking a solutions-focused approach that supports in-person networking, matching collaborators, and having dedicated support for coordination of activities. Our findings and those from other studies are helping to build the knowledge base and promote testable theories for promoting, enhancing, and sustaining team science.

### Limitations

It is important to note some limitations. First, the study was conducted with one group of implementation researchers whose work largely focused on behavioral health or substance use issues. However, this concern is mitigated by the fact that the D&I research of the group is fundamentally multidisciplinary in nature and spans diverse issues and settings. For example, issues addressed by faculty and fellows include mental health in emergency departments, behavioral issues related to gastrointestinal disease, testing multilevel implementation strategies in a range of service settings, implementation of evidence-based interventions in low- and middle-income countries, applying system dynamics and engineering approaches to D&I and use of health technologies. Related, while the participants represented a diverse array of disciplines, we acknowledge that not all disciplines (e.g., econometrics) were represented. Second, we did not use ancillary or other methods of data collection such as surveys. Thus, the results may not have identified all possible issues that would address team science in D&I research. However, the methods utilized allowed for participants to raise issues beyond what might have been included in a survey. Finally, we did not capture the degree to which responses were related to participants’ own use of strategies to promote team science versus strategies they had observed others using. However, members of team research initiatives can be and often are involved in creating, employing, and participating in team science promotion strategies, allowing for scoping observations from a wide variety of perspectives.

### Conclusions

Team science is being called for and increasingly expected by funders and academic institutions to facilitate collaboration, innovation, and productivity in regard to translating health innovations to real-world settings. There is a strong argument for using team science in D&I research because of its inherently broad and complex nature. Thus, it is important to understand and develop approaches for fostering and improving team science in D&I research. Our study results indicate that key strategies for fostering team science in D&I include (1) setting clear expectations and roles in conducting research, (2) promoting and modeling effective communication, and (3) establishing shared goals and mission. Pursuing these strategies will likely improve team cohesion and innovation, and result in approaches to accelerate the adoption, implementation, and sustainment of health innovations across contexts and with the promise of improving quality of care and outcomes.
